# Acute miliary tuberculosis in pregnancy after in vitro fertilization and embryo transfer: a report of seven cases

**DOI:** 10.1186/s12879-021-06564-z

**Published:** 2021-09-06

**Authors:** Xiaoyan Gai, Hongbin Chi, Wenli Cao, Lin Zeng, Lixue Chen, Weixia Zhang, Donghong Song, Ying Wang, Ping Liu, Rong Li, Yongchang Sun

**Affiliations:** 1 Department of Respiratory and Critical Care Medicine, Peking University Third Hospital, 49 N Garden Rd, Haidian District, Beijing, 100191 China; 2 Center for Reproductive Medicine, Department of Obstetrics and Gynecology, Peking University Third Hospital, 49 N Garden Rd, Haidian District, 100191 Beijing, China; 3 Tuberculosis Department, Beijing Geriatric Hospital, 102699 Beijing, China; 4 Clinical Epidemiology Research Center, Peking University Third Hospital, 100191 Beijing, China; 5 National Clinical Research Center for Obstetrics and Gynecology, 100191 Beijing, China; 6 Key Laboratory of Assisted Reproduction (Peking University), Ministry of Education, 100191 Beijing, China; 7 Beijing Key Laboratory of Reproductive Endocrinology and Assisted Reproductive Technology, 100191 Beijing, China

**Keywords:** Miliary tuberculosis, Infertility, In vitro fertilization, Embryo transfer

## Abstract

**Background:**

While miliary tuberculosis (TB) in pregnancy is rare after in vitro fertilization and embryo transfer (IVF-ET), it poses a serious threat to the health of pregnant women and their fetuses. The present study aimed to describe the clinical features of miliary TB and pregnancy outcomes of patients after IVF-ET.

**Methods:**

Data of infertile patients who received IVF-ET at Peking University Third Hospital between January 2012 and December 2017 were retrospectively analyzed. Patients who developed miliary TB during pregnancy were identified, and clinical characteristics of miliary TB were described.

**Results:**

Out of 62,755 infertile women enrolled, 7137 (11.4 %) showed signs of prior pulmonary TB on chest X-ray (CXR). Among the 15,136 women (mean age: 33.2 ± 5.0 years) who successfully achieved clinical pregnancy, seven patients aged 28–35 years had miliary TB during pregnancy, with two patients having a complication of TB meningitis. All these patients presented with fever. Notably, old TB lesions were detected on CXR in six patients before IVF-ET; nevertheless, no anti-TB therapy was administered. Furthermore, salpingography revealed oviduct obstruction in all patients (7/7). Patients received anti-TB therapy following a diagnosis of miliary TB and were clinically cured. However, pregnancy was terminated due to spontaneous (4/7) and induced (3/7) abortion.

**Conclusions:**

TB reactivation, mostly as miliary TB and TB meningitis, is severe in pregnant women after IVF-ET and deleterious to pregnancy outcomes. Signs of prior TB on CXR may be risk factors for TB reactivation during pregnancy.

## Background

Tuberculosis (TB) remains a major public health problem globally and poses a considerable threat to human health [1]. Globally, approximately 3.2 million women suffer from clinical TB each year [2]. Pregnancy-related TB endangers the health of both women and their fetuses and is considered an important cause of morbidity and mortality [3–5]. TB more rapidly progresses in pregnant women than in nonpregnant ones [5] and can lead to miscarriage [3–5]. Furthermore, women who survived from TB are often dissuaded from having children or, even worse, can no longer conceive again.

Acute miliary TB, a more serious and potentially lethal form of the disease, results from massive hematogenous dissemination of *Mycobacterium tuberculosis*. The miliary pattern in the lungs has been radiologically described as “a collection of tiny discrete pulmonary opacities that are generally uniform in size and widespread in distribution, each of which measures 2 mm or less in diameter” [6, 7]. If untreated, miliary TB is uniformly fatal. Relative to all forms of TB, the incidence of miliary TB ranges from 0.15 to 10 % [1, 6, 7]. Additionally, approximately 15–30 % of patients with pulmonary TB during pregnancy exhibit hematogenous dissemination and have miliary TB [8]. Because clinical symptoms such as fever and cough are nonspecific and chest X-ray (CXR) and chest computed tomography (CT) scan during pregnancy are associated with a risk of radiation exposure, the diagnosis of miliary TB during pregnancy is often delayed.

With the increasing application of in vitro fertilization and embryo transfer (IVF-ET), the incidence of TB during pregnancy has gradually increased, posing a serious threat to the health of pregnant women and fetuses [1, 9, 10]. There have been occasional case reports of TB with hematogenous dissemination, miliary TB, and/or meningitis during pregnancy after IVF-ET, leading to abortion, fetal malformation, or increased risk of mortality [10, 11]. Therefore, correct and timely diagnosis and management of TB during pregnancy are important. Therefore, in this study, we aimed to describe the clinical features of TB and its impact on pregnancy outcomes after IVF-ET. We retrospectively analyzed the data of patients who underwent IVF-ET and showed clinical signs of miliary TB during pregnancy between January 2012 and December 2017 at the reproductive center of our hospital. Additionally, we summarized the clinical manifestations and pregnancy outcomes of these patients.

## Methods

This was a retrospective study of patients who underwent IVF-ET for infertility between January 1, 2012, and December 31, 2017, at Peking University Third Hospital, a tertiary referral hospital in Beijing, China. Data on patients undergoing IVF-ET, including causes of infertility, serum hormone concentrations, the controlled ovarian hyperstimulation protocol, and CXR results, were recorded. CXR was routinely performed for each patient, and active TB cases were excluded before IVF-ET was started. A medical team was assigned to follow up the pregnancy outcomes.

During the 6-year period, 62,755 patients, who were all HIV-negative, had received IVF-ET at our center. Among these patients, seven with active TB during pregnancy were identified. Active TB was diagnosed according to the national guidelines [12]. Miliary TB was diagnosed based on the size, distribution, and density of miliary-like nodules that were bilaterally distributed on CXR or chest CT scan [13, 14]. Baseline data and CXR and laparoscopy results before IVF-ET were retrieved. A respiratory physician contacted the seven patients via phone call and reviewed the medical records. Live birth was defined as the delivery of a living fetus (or living fetuses) beyond 28 weeks of gestation, whereas miscarriage was defined as pregnancy loss before 28 weeks of gestation.

This study was approved by the Ethics Committee of Peking University Third Hospital [batch number: (2019)327-02]. The retrospective nature of the study resulted in a waiver regarding the signing of the informed consent form.

### IVF-ET protocol

IVF-ET was performed as previously described [15]. Briefly, controlled ovarian hyperstimulation was achieved, oocytes were fertilized, and ETs were subsequently performed [15]. Among seven patients who developed TB during pregnancy, one had undergone a frozen cycle transfer, whereas the remaining six had undergone fresh cycle transfer. After ET, 60 mg of progesterone was injected intramuscularly for 14 days. Blood human chorionic gonadotropin concentration was monitored at 2 weeks after transplantation, and the status of the embryo sac was examined by ultrasonography at 4 weeks after transplantation.

### Statistical analysis

Continuous variables are expressed as mean ± standard deviation or as median with interquartile range. Statistical analysis was performed using SPSS version 23 (IBM Corp., Armonk, NY).

## Results

### Patients’ baseline data

**Fig. 1:**
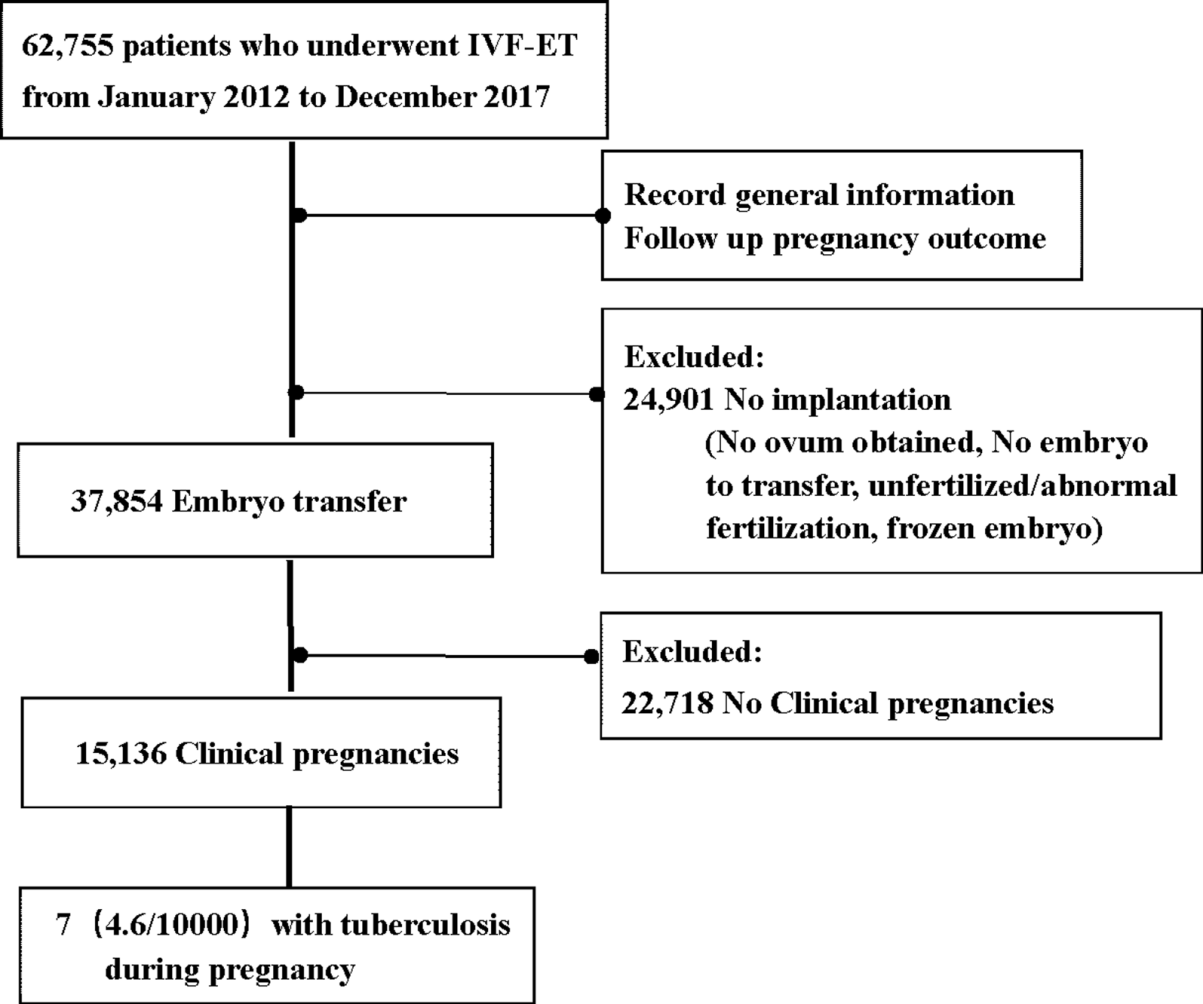
Flow of protocol and outcomes

A total of 62,755 infertile patients (mean age: 33.1 ± 5.1 years, range: 20–50 years) were identified from our database to have been referred to the reproductive center of our hospital between January 1, 2012, and December 31, 2017. Of these patients, 11.4 % (7137/62,755) exhibited signs of prior pulmonary TB prior to IVF-ET based on their CXR results. Furthermore, 37,854 out of all 62,755 patients underwent ET, whereas the remaining 24,901 patients failed because they had no ovum that could be obtained or had no embryo to transfer or due to some other reasons. Finally, 15,136 (mean age: 33.2 ± 5.0 years) out of 37,854 patients succeeded in achieving clinical pregnancy. Among these 15,136 patients, seven had acute miliary TB during pregnancy. Hence, the prevalence rate was 7/15,136 (i.e., 4.6/10,000) (Fig. 1).

### Baseline data of the seven patients with active TB during pregnancy

**Fig. 2:**
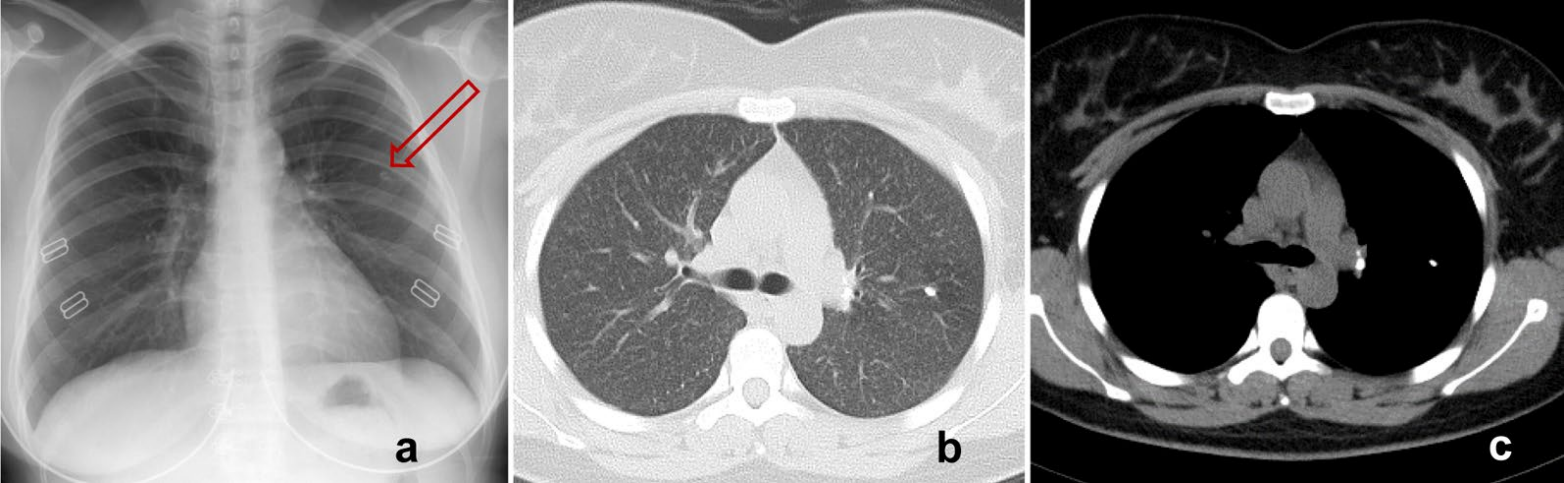
Chest imaging in a 32-year-old infertile woman. **a** Posteroanterior chest radiograph showing a scarring nodule in the left upper lobe (arrow) at screening before IVF-ET. **b** Chest computed tomography showing multiple miliary nodules of uniform density, size, and distribution. **c** A scarring nodule in the left upper lobe and calcification of the left hilar lymph nodes. IVF-ET, in vitro fertilization and embryo transfer

Among the seven cases, four occurred in 2012, two in 2016, and one in 2017. These pregnant women were between 28 and 34 years of age, and the duration of infertility ranged from 1 to 12 years. Their body mass index was 20.1–27.3 kg/m^2^. All seven patients had primary infertility due to unilateral or bilateral oviduct obstruction, as assessed using salpingography. Four patients also underwent laparoscopy and showed tubal obstruction and adhesion consistent with TB; however, the pathology failed to reveal features of TB. One patient had suffered from TB at the age of 16, and the local hospital administered anti-TB therapies for over 6 months at that time. The other six patients had no clinical history of TB and had not received anti-TB treatment. Among the seven patients, six showed signs of old pulmonary TB lesions on CXR before IVF-ET. Tuberculin skin test (TST) was performed in three patients before IVF-ET, with induration diameters of 10–20 mm, thus confirmed as positive (+ +) cases. However, this test was not performed in the other four patients. None had active TB before IVF-ET, and IVF-ET was performed as scheduled (Fig. 2; Table 1).

**Table 1:** Baseline characteristics of the seven cases of miliary TB

Case	Type of infertility	Infertility duration (years)	Infertility factor	Past history	CXR before IVF-ET	Salpingography	Laparoscopy	Pathology	ESR
1	Primary infertility	12	Fallopian tubal ovulation	Denial of TB history	Fibrotic scars in upper left lung	Bilateral oviduct obstruction	Extensive pelvic adhesion, bilateral ovarian adhesion. Yellow hard neoplasm could be seen at the umbrella end of the right oviduct and the mesentery; Pelvic adhesiolysis and salpingostomy;	Peritoneal fibrous nodules complicated by hyaline degeneration	8
2	Primary infertility	4	Fallopian tubal ovulation	Denial of TB history	Left pleural thickening and adhesion	Bilateral oviduct umbrella end adhesion	Bilateral oviduct obstruction, after laparoscopic recanalization	Endometritis	6
3	Primary infertility	9	Fallopian tubal ovulation	Denial of TB history	Fibrotic scars in upper right lung	Bilateral oviduct obstruction	NA	NA	6
4	Primary infertility	7	Fallopian tubal ovulation	Denial of TB history	Fibrotic scars in upper left lung	Bilateral oviduct obstruction	Bilateral oviduct obstruction; Bilateral salpingoplasty	manifestation of secretive phase	6
5	Primary infertility	5	Fallopian tubal ovulation; PCOS	Denial of TB history	No abnormality	Left oviduct obstruction; Right oviduct unsmooth;	Bilateral oviduct obstruction	Endometritis	5
6	Primary infertility	1	Fallopian tubal ovulation	Denial of TB history; One IVF-ET failed history;	Left pleural thickening	Left oviduct obstruction; Right oviduct unsmooth;	NA	NA	5
7	Primary infertility	3	Fallopian tubal ovulation	Pulmonary TB at the age of 16	Fibrous scars in the upper right lung; Left pleural thickening and adhesion	Bilateral oviduct obstruction	NA	NA	6

*CXR* chest X-ray, *IVF-ET *in vitro fertilization and embryo transfer, *TB* tuberculosis, *ESR* erythrocyte sedimentation rate

One patient underwent frozen ET and had a singleton pregnancy. The remaining six patients underwent fresh ET: three had twin pregnancies and three had singleton pregnancies.

### Clinical manifestations and diagnosis of active TB during pregnancy

All seven patients had fever at 7–14 weeks of pregnancy. Among them, six had moderate-to-high fever, with the highest body temperatures recorded at 38.5–40 °C, whereas one had low-grade fever (37.5 °C). All seven patients had mild cough and a small amount of sputum with (1/7) or without blood (6/7). CXR and CT scans were performed on all seven patients, which showed diffuse miliary nodules in both lungs, consistent with acute miliary TB (Fig. 2). Two patients with complaints of significant headache were confirmed by lumbar puncture to have TB meningitis (Table 1).

Five patients underwent an interferon gamma release assay (IGRA) test after fever onset and showed positive results. One patient underwent the TST, which was positive (+ + +).

### Outcomes of TB and pregnancy outcomes of the seven patients

After the diagnosis of TB, four patients had spontaneous abortion, whereas three patients underwent induced abortion (Table 2). All seven patients recovered after anti-TB therapy. At follow-up, two patients achieved pregnancy after second IVF-ET.

**Table 2:** Clinical manifestations and pregnancy outcomes of the seven cases of miliary TB during pregnancy

Case	IVF-ET cycle type	IVF-ET outcomes	Vaginal bleeding	Gestational weeks with bleeding (w)	Fever	Gestational weeks with fever (w)	TST after fever onset	IGRA after fever onset	Pregnancy outcomes	Follow-up
1	Fresh ET	Singleton	+	7	+	12	NA	NA	Spontaneous abortion	Received ET again and gave birth 3 years later after TB.
2	Fresh ET	Twin	+	17	+	14	NA	+	Spontaneous abortion	Underwent two rounds of ET 3 years later, yet both failed.
3	Fresh ET	Singleton	−	−	+	9	NA	+	Induced abortion	No pregnancy since.
4	Fresh ET	Twin	−	−	+	7	NA	+	Induced abortion	Underwent ET 3 years later yet failed. No pregnancy since.
5	Frozen ET	Singleton	+	8	+	10	+ + +	+	Spontaneous abortion	No pregnancy after three rounds of ET.
6	Fresh ET	Twin	+	14	+	12	NA	+	Spontaneous abortion	Underwent fresh ET and two rounds of frozen ET 5 years later, yet all failed. No pregnancy since.
7	Fresh ET	Singleton	+	8	+	9	NA	NA	Induced abortion	Experienced one failed frozen ET 6 years later; got pregnant and gave birth after another ET 7 years later.

*IVF-ET *in vitro fertilization and embryo transfer, *TB* tuberculosis, *ET* embryo transfer, *TST* tuberculin skin test, *IGRA* interferon gamma release assay, *NA* not available

## Discussion

In this retrospective study, we identified seven cases of active TB during pregnancy from 62,755 cases of IVF-ET carried out at our hospital. All of these seven cases were diagnosed with acute miliary TB, with two cases complicated by TB meningitis. Notably, signs of prior TB on CXR were detected in 11.4 % of our study population (7137/62,755), and six out of the seven patients with acute miliary TB had prior TB signs identified on CXR before IVF-ET.

Our data indicated that TB in pregnancy after IVF-ET mostly occurred during the first 8–12 weeks of pregnancy. Fever was the main symptom, and the time interval between fever onset and definitive diagnosis was 2–4 weeks or more. An important finding of our study was that all seven patients with active TB during pregnancy after IVF-ET developed hematogenous dissemination, which is the most serious condition of TB. Two out of the seven patients had tuberculous meningitis as a complication. This finding is consistent with the result of a previous report. We conducted a literature review on patients with TB during pregnancy after IVF-ET using the keywords “infertility,” “in vitro fertilization and embryo transfer,” “tuberculosis,” and “pregnancy” to search for articles published from 1980 to 2019 in PubMed, MEDLINE, EMBASE, and Chinese Wanfang databases. Furthermore, we summarized 37 cases of TB during pregnancy after IVF-ET [10, 16–24] (Table 3). Addis et al. reported the first case in 1988 [10]. Since then, more cases have been described, the majority of which were from developing countries, [17–21, 24]. The results from our study and from these previous studies indicated that women with TB during pregnancy after IVF-ET were prone to hematogenous dissemination.

**Table 3:** Summary of reported cases of TB during pregnancy after IVF-ET

Cases	Age (years)	Onset time (week)	TB history	Clinical manifestations	Diagnosis	Pregnancy outcomes for pregnant women	Outcomes for fetus	Country	References
1	33	10	Denial of TB history	Fever, cough, shortness of breath	Miliary TB (1/1)	Cured	Spontaneous abortion	U.K.	Addis et al. [10]
5	25–33	5–9	Denial of TB history; Laparoscopy showed bilateral oviduct obstruction (5/5)	Fever (5/5)	Miliary TB (5/5)	Cured	Spontaneous abortion (5/5)	China	Wei et al. [16]
4	NA	5–15	Denial of TB history	Fever (4/4)	Miliary TB (4/4); ARDS (1/4)	Died (1/4); Cured (3/4)	Spontaneous abortion (4/4)	China	Wei et al. [17]
1	29	11	Denial of TB history; laparoscopy showed bilateral oviduct obstruction	Fever, shortness of breath	Miliary TB (1/1)	Cured	Spontaneous abortion	China	Liu et al. [18]
6	27–32	6–9	One case had a history of tuberculous pleuritis, and 1 case had a history of pelvic TB	Fever (6/6), slight cough and expectoration (6/6)	Miliary TB (6/6)	Cured	Spontaneous abortion (5/6); induced abortion (1/6)	China	Gao et al. [19]
11	26–36	6–14	Denial of TB history	Fever (11/11)	Miliary TB (11/11); TB meningitis (4/11)	Cured	Spontaneous abortion (8/11); induced abortion (3/11)	China	Jin et al. [20]
6	29–39	5–16 (5/6); 26 (1/6)	One case had a history of TB, one case had no history of TB, but chest radiograph showed sclerotic calcification in the lung(s), and the other 4 cases had no manifestation of TB	Fever, cough, shortness of breath (6/6); Headache (1/6)	Miliary TB (6/6); TB meningitis (1/6)	Cured	Spontaneous abortion (3/6); induced abortion (3/6)	China	Ye et al. [21]
1	38	14	Denial of TB history, and laparoscopy showed bilateral oviduct obstruction	Fever, cough	Miliary TB with TB meningitis (1/1)	Cured	Spontaneous abortion	Israel	Gull et al. [22]
1	NA	8	Denial of TB history; Laparoscopy showed bilateral oviduct obstruction	Fever, cough	Miliary TB (1/1)	Cured	Spontaneous abortion	Belgium	Jacquemyn et al. [23]
1	31	8	Denial of TB history; Laparoscopy showed bilateral oviduct obstruction	Fever, cough	Miliary TB (1/1)	Cured	Premature delivery	China	Fan et al. [24]

*TB* tuberculosis, *IVF-ET *in vitro fertilization and embryo transfer, *ARDS* acute respiratory distress syndrome

TB activation and dissemination may be related to latent infection, IVF-ET intervention, and immune dysregulation in pregnancy [25]. Studies have shown that estrogen, progesterone, and human chorionic gonadotropin have a direct inhibitory effect on T-cells [26, 27]. High estrogen levels are conducive to the proliferation of *M. tuberculosis*. Increased vascular permeability after pregnancy may also facilitate bacterial spread throughout the body, resulting in hematogenous dissemination [28]. The prognosis of miliary TB during pregnancy after IVF-ET was poor and may have caused the spontaneous abortion or may have resulted in premature delivery. More seriously, respiratory failure and even acute respiratory distress syndrome might occur in pregnant women [14, 16]. Furthermore, fetuses might suffer from intrauterine growth retardation or be stillborn due to hypoxia, or acquire infection via hematogenous dissemination or aspiration of contaminated amniotic fluid [11]. Moreover, those with miliary TB during pregnancy were less likely to achieve pregnancy, even with IVF-ET.

Identifying patients at high risk for TB activation should be an important evaluation before IVF-ET, especially in regions with a high TB burden. From our observation, we speculate that the coexistence of primary infertility, untreated prior pulmonary TB, and fallopian tube obstruction may be a risk factor for active TB during an IVF-ET pregnancy. Signs of fibrotic scarring, calcified nodules, and/or pleural thickening on CXR indicate previous infection with *M. tuberculosis* [29–31]. In our series, among the 7137 patients who had “old TB” lesions on CXR, six developed miliary TB during pregnancy. Liu et al. reported a similar case in which untreated prior pulmonary TB developed into miliary TB during pregnancy [18]. Our previous study revealed that the clinical pregnancy and live birth rates were significantly lower in infertile patients with untreated prior pulmonary TB than in those without signs of prior TB, highlighting the adverse effects of TB in this specific patient population [32].

Genital TB (GTB) is a form of extrapulmonary TB and a major cause of primary infertility among women in TB-endemic countries [33], with a prevalence rate of 28.4 % in our hospital, as observed in previous studies [34, 35]. GTB may cause fallopian tube obstruction, reduced endometrial receptivity, and ovarian dysfunction, leading to infertility. However, manifestations of GTB are nonspecific, and confirmation of diagnosis relies on invasive procedures. Our seven patients showed unilateral or bilateral oviduct obstruction, which suggested chronic infections such as GTB. Further studies are required to clarify whether the IGRA test and TST are necessary for the assessment of latent TB infection prior to IVF-ET and whether preventive anti-TB therapy can improve the pregnancy outcomes of infertile women with latent TB infection or untreated prior pulmonary TB on CXR. Moreover, screening for latent TB infection during pregnancy can provide an excellent opportunity for prevention.

Imaging plays a pivotal role in the diagnosis of pulmonary diseases, including TB [31, 36]. Clinical diagnosis of active TB in pregnant women is often delayed, which is largely attributable to the concern about radiation exposure from chest radiography. The IGRA test is an important diagnostic method for active TB detection and is safe for use during pregnancy [37–40]. Both the IGRA test and TST have a high consistency of 77.3–88.0 % [39]. The IGRA test has a high sensitivity of 100% and a moderate specificity of 80.0% for detecting active TB during pregnancy [40], which are not affected by previous vaccination with bacillus Calmette–Guérin. Further studies on the use of the IGRA test for TB detection during pregnancy, particularly in high-risk patients from TB-endemic regions, are warranted.

Our study has some limitations. First, this was a single-center study; however, as the largest reproductive center in China, we perform more than 10,000 cycles of IVF-ET annually on women from all over the country. Therefore, the population in this study was representative. Second, we inquired whether active TB had occurred during pregnancy through telephone follow-up, which might have led to an underdiagnosis of the disease.

## Conclusions

Acute miliary TB occurs in pregnant women after IVF-ET, particularly in those exhibiting signs of prior pulmonary TB on CXR. Patients with miliary TB have poor pregnancy outcomes. The coexistence of primary infertility, untreated prior pulmonary TB, and fallopian tube obstruction is a high-risk factor for TB dissemination. Therefore, clinicians should be aware of the signs of TB before administering a course of IVF-ET treatment. Prospective studies are warranted to determine the incidence of and risk factors for reactive TB in infertile patients with prior pulmonary TB after IVF-ET and to clarify whether anti-TB therapy is beneficial for pregnancy outcomes in these patients.

## Data Availability

The datasets used and/or analyzed during the current study are available from the corresponding author on reasonable request.

## Abbreviations

CT: Computed tomography
CXR: Chest X-ray
GTB: Genital TB
IGRA: Interferon gamma release assay
IVF-ET: In vitro fertilization and embryo transfer
TB: Tuberculosis
TST: Tuberculin skin test
